# Inhibitory Effect of Inflexinol on Nitric Oxide Generation and iNOS Expression via Inhibition of NF-*κ*B Activation

**DOI:** 10.1155/2007/93148

**Published:** 2007-04-03

**Authors:** Jae Woong Lee, Moon Soon Lee, Tae Hun Kim, Hwa Jeong Lee, Seong Su Hong, Young Hee Noh, Bang Yeon Hwang, Jai Seup Ro, Jin Tae Hong

**Affiliations:** ^1^College of Pharmacy and CBITRC, Chungbuk National University 12, Gaesin-dong, Heungduk-gu, Cheongju, Chungbuk 361-763, South Korea; ^2^National Institute of Environmental Research, Kyungseo-dong, Seo-gu, Incheon 404-780, South Korea; ^3^Department of Medical Beauty, Konyang University, 26 Nae-dong, Nonsan, Chungnam 320-711, South Korea

## Abstract

Inflexinol, an *ent*-kaurane diterpenoid, was isolated from the leaves of *Isodon excisus*. Many diterpenoids isolated from the genus *Isodon* (*Labiatae*) have antitumor and antiinflammatory activities. We investigated the antiinflammatory effect of inflexinol in RAW 264.7 cells and astrocytes. As a result, we found that inflexinol (1, 5, 10 *μ*M) suppressed the expression of inducible nitric oxide synthase (iNOS) and cyclooxygenase-2 (COX-2) as well as the production of nitric oxide (NO) in LPS-stimulated RAW 264.7 cells and astrocytes. Consistent with the inhibitory effect on iNOS and COX-2 expression, inflexinol also inhibited transcriptional and DNA binding activity of NF-*κ*B via inhibition of I*κ*B degradation as well as p50 and p65 translocation into nucleus. These results suggest that inflexinol inhibits iNOS and COX-2 expression through inhibition of NF-*κ*B activation, thereby inhibits generation of inflammatory mediators in RAW 264.7 cells and astrocytes, and may be useful for treatment of inflammatory diseases.

## 1. INTRODUCTION

Nitric oxide (NO), free radical produced by the inducible NO synthase (iNOS) isoform, is an essential component of the host innate immune and inflammatory response to a variety of pathogens [1]. It has diverse physiological roles and may also contribute towards pathological processes. When NO is synthesized in large quantities by activated inflammatory cells, it has cytotoxic properties and may be involved in the pathogenesis of acute and chronic inflammatory conditions [2]. In particular, NO is claimed to contribute to damage of the joint cartilage in rheumatoid arthritis, to mucosal injury in inflammatory bowel disease and to degeneration of neurons in neurodegenerative diseases such as multiple scleroses, Parkinson's disease, and Alzheimer's disease [3]. In most neurodegenerative disorders, a massive neuronal cell death occurs as a consequence of an uncontrolled inflammatory response, where activated astrocytes and microglia and their cytotoxic agents play a crucial pathological role [4]. Glial cells consisting of astrocytes and microglia can produce cytokines, reactive oxygen radicals, and NO in response to ischaemic, traumatic, and infectious insults, leading to exaggeration of the disease processes [5].

Coinduction or coregulation of cyclooxygenase-2 (COX-2) and iNOS have been demonstrated in a number of cell culture studies and animal inflammatory model [6, 7]. Both COX-2 and iNOS are inducible form of enzymes up-regulated in response to inflammation challenge. In inflammatory process, COX-2 is expressed in many cells including fibroblast and macrophages, and produces prostaglandins that contribute to the pain and swelling of inflammation [8].

Expression of these inflammatory genes such as iNOS and COX-2 can be regulated by the activation of the nuclear factor-*κ*B (NF-*κ*B). Research of the literature reveals that there are one NF-*κ*B consensus DNA sequence within COX-2 promoter [9], and two NF-*κ*B DNA consensus sequences within iNOS promoter [10] that are responsible for LPS-induced NF-*κ*B DNA binding activity. The most common active form of the NF-*κ*B family is the p50/p65 or p52/p65 heterodimer. In most cell types, inactive NF-*κ*B complexes are sequestered in the cytoplasm via their noncovalent interaction with inhibitory proteins known as I*κ*Bs. In response to multiple stimuli, including cytokines, virus, and stress-inducing agents, the latent cytoplasmic NF-*κ*B/I*κ*B*α* complex is activated by phosphorylation on conserved serine residues in the *N*-terminal portion of I*κ*B. After that, activated NF-*κ*B translocates to the nucleus and binds to its cognate DNA binding site in the promoter or enhancer regions of specific genes [11]. NF-*κ*B is a major transcription factor that plays an essential role in several aspects of human health including the development of inflammation and immunity [12]. The dysregulation of NF-*κ*B is associated with many disease states such as atherosclerosis, arthritis, cancer. Therefore, appropriate regulation and control of NF-*κ*B activity would provide a potential approach for the management of NF-*κ*B-related human diseases [11].

*Isodon excisus*, named *Oh Ri Bang Pul* in Korea, belongs to the genus *Isodon* and is distributed in Korea and Japan. The extracts have been used in fork medicine in Korea for treating a bruise, inflammation and pain. The genus *Isodon* (also called *Rabdosia*) is a rich source of diterpenes, especially the highly oxidized kaurene diterpenes. *Ent*-kaurene is the main diterpene intermediate involved in the biosynthesis of gibberellins, a widespread family of plant hormones with isoprenoid structure that control various physiological plant functions such as growth, germination, and flowering [13]. Some kaurene ditrerpene compounds have been demonstrated to exhibit not only cytotoxic activity against various cancer cell lines but also inhibitory activity of the NF-*κ*B pathway in macrophages [14–16]. For example, Linearol, a kaurene diterpene that impaired the inflammatory signaling by inhibiting NF-*κ*B inducing kinase in LPS-induced J774 macrophages [17]. Kamebakaurin, another kaurane diterpene also inhibited TNF-*α*-induced NF-*κ*B activation by direct covalent modification of cysteine 62 in the p50 in MCF-7 cells [18]. Therefore, much interest has recently been shown in the biological effects of kaurene diterpenes.

In the present study, we investigated antiinflammatory activity of inflexinol and its possible mechanisms in cultured RAW 264.7 cells and astrocytes. Inflexinol inhibited LPS-induced NO production as well as LPS-induced expression of iNOS and COX-2 in RAW 264.7 cells and astrocytes. Using gel shift assay and NF-*κ*B luciferase assay, we showed that inflexinol inhibited activation of the transcriptional factor NF-*κ*B, a central regulator of iNOS and inflammatory response of body. These our data provide evidence that inflexinol has inhibitory effect on NO production through inhibition of NF-*κ*B activation. These results suggest that inflexinol can be used for an antiinflammatory agent.

## 2. MATERIALS AND METHODS

### 2.1. Chemicals and reagents

**Figure 1:**
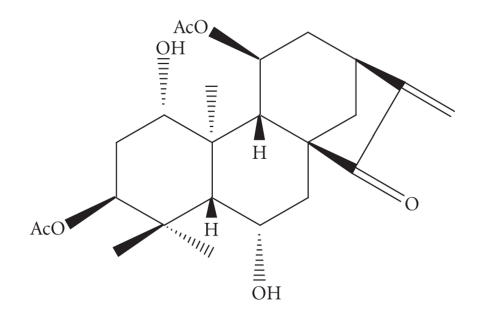
Chemical structure of inflexinol (*ent*-1*β*,3*α*,6*β*,11*α*-tetrahydroxykaur-16-ene-15-one 3,11-diacetate).

Inflexinol (Figure 1) was isolated from *Isodon excisus* (*Labiatae*). The dried aerial parts of *I. excisus* (1.6 kg) were pulverized and extracted with MeOH (3 × 1.5 L) at room temperature (24 hours). The extract was filtered and concentrated, in vacau and suitably diluted with water, then partitioned with n-hexane (3 × 1.5 L) and CH_2_Cl_2_ (3 × 1.5 L), respectively. The CH_2_Cl_2_ extract (13.7 g) was subjected to column chromatography on silica gel (9 × 25 cm, 70–230 mesh) eluting with n-hexane-acetone (5 : 1, 3 : 1, 3 : 2, acetone) affording five fractions (IEC-1 ∼ IEC-5). Fraction IEC-3 was subjected to flash column chromatography on RP-18 (2 × 30 cm, 40–63 *μ*m) eluting with CH_3_CN : H_2_O (30 : 70) and semiprepatative HPLC (column: YMC-ODC, 20 × 150 mm) eluting with CH_3_CN : H_2_O (23 : 77) at the flow speed of 6.5 mL/min. The structure of this compound was determined as *ent*-1*β*,3*α*,6*β*,11*α*-tetrahydroxykaur-16-ene-15-one-3,11-diacetate (inflexinol), by comparison of its physicochemical and spectral data with those of literature [19].

LPS was obtained from Sigma Aldrich (St Louis, Mo, USA) Dulbecco's modified Eagle's medium (DMEM), fetal bovine serum, penicillin, and streptomycin were purchased from Invitrogen (Carlsbad, Calif, USA).

### 2.2. RAW 264.7 cell culture

RAW 264.7 cells were obtained from the American Type Culture Collection (Rockville, Md, USA). These cells were maintained at subconfluence in a 95% air, 5% CO_2_ humidified atmosphere at 37°C. The medium used for routine subcultivation was Dulbecco's Modified Eagle's Medium (DMEM, Invitrogen, Carlsbad, Calif, USA), supplemented with 10% fetal bovine serum (FBS), penicillin (100 units/mL), and streptomycin (100 *μ*g/mL). Cells were counted with a hemocytometer and the number of viable cells was determined through trypan blue dye exclusion.

### 2.3. Astrocyte culture

The Sprague-Dawley rats were maintained in accodance with the policy of the National Institute of Toxicological research, which is in accord with the Korea Food and Drug Administration's guideline for the care and use of laboratory animals. Sprague-Dawley rats weighing 200–300 g were housed under 12-hour light/dark cycles, at 23°C, and 60 ± 5% humidity. All animals had free access to food (Samyang Foods, Seoul, South Korea) and water. Cerebral cortical cells were isolated from neonatal rat brain (day 1) in PBS (0.1 mol). After washing with Dulbecco's modified Eagle's medium (DMEM), the isolated cells were incubated for 15 minutes in DMEM containing 0.2% trypsin. Cells were dissociated by trituration and plated into polyethyleneimine-coated plastic (5 × 10^5^ cells/60 mm dish) containing minimum essential medium with Eagle's salts supplemented with 10% heat-inactivated fetal bovine serum, 2 mM L-glutamine, 1 mM pyruvate, 20 mM KCl, 10 mM sodium bicarbonate, and 1 mM Hepes (pH 7.2). After 3 days in culture, the culture medium was replaced with DMEM containing 10% fetal bovine serum, and medium was changed every 3 days of culture. Cells were cultured for designated time. The cultured cells contained <10% neuronal cells.

### 2.4. Cell viability assay

The cytotoxicity of inflexinol was evaluated using the WST-8 assay (Dojindo Laboratories, Tokyo, Japan). WST-8 [2-(2-methoxy-4-nitrophenyl)-3-(4-nitrophenyl)-5-(2,4-disulfophenyl)-2H-tetrazolium, monosodium salt] is reduced by dehydrogenases in cells to give a yellow-colored product (formazan), which is soluble in the culture medium. The amount of the formazan dye generated by the activity of dehydrogenases in cells is directly proportional to the number of living cells. In brief, 1 × 10^4^ cells per well were plated into 96-well plates, incubated at 37°C for 24 hours, and given a fresh change of medium. Cells were then incubated with or without LPS (1 *μ*g/mL) in the absence or presence of various concentrations of inflexinol at 37°C for an additional 24 hours. At that point, 10 *μ*l of the WST-8 solution were added to the wells and incubation was continued for another 1 hour. The resulting color was assayed at 450 nm using a microplate absorbance reader (Sunrise, Tecan, Switzerland).

### 2.5. Nitrite assay

Cells were grown in 96-well plates and then incubated with or without LPS (1 *μ*g/mL) in the absence or presence of various concentrations of inflexinol for 24 hours. The nitrite accumulation in the supernatant was assessed by Griess reaction [20]. Each 50 *μ*l of culture supernatant was mixed with an equal volume of Griess reagent [0.1% N-(1-naphthyl)-ethylenediamine, 1% sulfanilamide in 5% phosphoric acid] and incubated at room temperature for 10 minutes. The absorbance at 540 nm was measured in a microplate absorbance reader, and a series of known concentrations of sodium nitrite was used as a standard.

### 2.6. Western blot analysis

Cells were homogenized with protein extraction solution (PRO-PREP, Intron Biotechnology, South Korea), and lysed by 40-minute incubation on ice. The lysate centrifuged at 15 000 rpm for 15 minutes. Equal amount of proteins (40 *μ*g) were separated on an SDS/10%-polyacrylamide gel, and then transferred to a polyvinylidene difluoride (PVDF) membrane (GE Water & Process technologies, Trevose, Pa, USA). Blots were blocked for 2 hours at room temperature with 5% (w/v) nonfat dried milk in Tris-buffered saline Tween-20 [TBST: 10 mM Tris (pH 8.0) and 150 mM NaCl solution containing 0.05% Tween-20]. After a short wash in TBST, the membrane was incubated at room temperature with specific antibodies. Rabbit polyclonal antibodies against iNOS and COX-2 (1 : 1000) (Cayman Chemical, Ann Arbor, Mich, USA), and rabbit polyclonal antibodies against p65 and I*κ*B*α* (1 : 500), and mouse monoclonal antibody against p50 (1 : 500) (Santa Cruz Biotechnology Inc. Santa Cruz, Calif, USA) were used in study. The blot was then incubated with the corresponding conjugated antirabbit or mouse immunoglobulin G-horseradish peroxidase (Santa Cruz Biotechnology Inc. Santa Cruz, Calif, USA). Immunoreactive proteins were detected with the ECL western blotting detection system.

### 2.7. Gel electromobility shift assay

Gel shift assays were performed according to the manufacturer's recommendations (Promega, Madison, Wis, USA). Briefly, 5 × 10^6^ cells was washed twice with 1× PBS, followed by the addition of 1 mL of PBS, and the cells were scraped into a cold Eppendorf tube. Cells were spun down at 13 000 rpm for 5 minutes, and the resulting supernatant was removed. Cells were suspended in 400 *μ*l of solution A containing 10 mM HEPES, pH 7.9, 1.5 mM MgCl_2_, 10 mM KCl, 0.5 mM dithiothreitol, 0.2 mM phenylmethylsulfonyl fluoride; vigorously vortexed; allowed to incubate on ice for 10 minutes; and centrifuged at 12 000 rpm for 6 minutes. The pelleted nuclei were resuspended in solution C (solution A + 420 mM NaCl, 20% glycerol) and allowed to incubate on ice for 20 minutes. The cells were centrifuged at 15 000 rpm for 15 minutes, and the resulting nuclear extract supernatant was collected in a chilled Eppendorf tube. Consensus oligonucleotides were end-labeled using T4 polynucleotide kinase and [*γ*-^32^P] ATP for 10 minutes at 37°C. Gel shift reactions were assembled and allowed to incubate at room temperature for 10 minutes followed by the addition of 1 *μ*l (50 000–200 000 cpm) of ^32^P end-labeled oligonucleotide and another 20 minutes of incubation at room temperature. Subsequently 1 *μ*l of gel loading buffer was added to each reaction and loaded onto a 6% nondenaturing gel and electrophoresis until the dye was four-fifths of the way down the gel. The gel was dried at 80°C for 1 hour and exposed to film overnight at 70°C.

### 2.8. Transfection and asssy of NF-*κ*B luciferase activity

RAW 264.7 cells and astrocytes were plated at a density of 1 × 10^5^ cells per 24-well plate. After 24 hours of growth to 90% confluence, the cells were transfected with pNF-*κ*B-Luc plasmid (5× NF-*κ*B; Stratagene, Calif, USA) using a mixture of plasmid and lipofectAMINE PLUS in OPTI-MEN according to manufacture's specification (Invitrogen, Carlsbad, Calif, USA). Luciferase activity was measured by using the luciferase assay kit (Promega, Madison, Wis, USA) according to the manufacturer's instructions (WinGlow, Bad Wildbad, Germany).

### 2.9. Statistical evaluation

The data represent the mean ± (SE) of three independent experiments performed in triplicate. Statistical analysis was performed by one-way ANOVA, followed by a Dunnett test as post hoc comparison. Differences were considered significant at *P* < .05.

## 3. RESULTS

### 3.1. Effect of inflexinol on cell viability in RAW 264.7 cells and astrocytes

**Figure 2:**
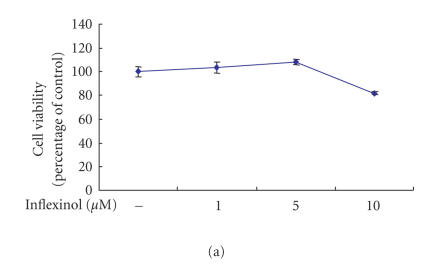

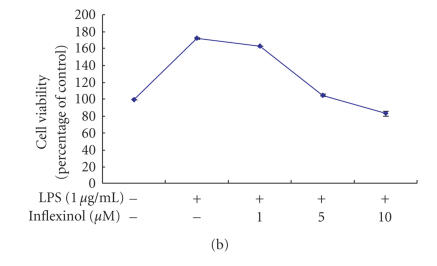

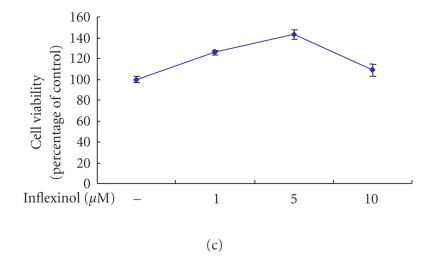

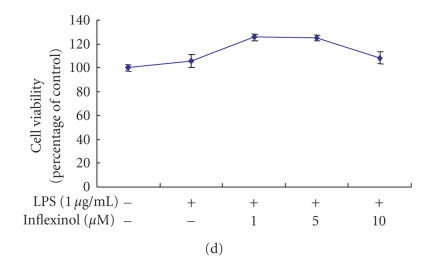
Effect of inflexinol on viability of RAW 264.7 cells (a) and (b) and astrocytes (c), (d). The cell viability was evaluated using a WST-8 assay. Cells were incubated with inflexinol in the absence of LPS (a) and (c) and incubated with inflexinol in the presence of LPS (b) and (d). Results were given in percent related to untreated controls. The data represent the mean ± (SE) of three independent experiments performed in triplicate.

After RAW 264.7 cells were incubated with inflexinol in the absence of LPS, inflexinol increased slightly cell viability at lower concentrations (1, 5 *μ*M) and showed mild reduction (<20%) of cell viability at highest concentration (10 *μ*M) used (Figure 2(a)). When RAW 264.7 cells were incubated with inflexinol in the presence of LPS, LPS remarkably increased the cell viability. Although mild reduction of cell viability (<20%) was showed by 10 *μ*M of inflexinol like previous case, it is considered that inhibitory effect of inflammatory mediators by infleixnol is not related with cytotoxic effect (Figure 2(b)).

Moreover, inflexinol (with or without LPS) did not decrease the cell viability at the various concentrations (1, 5, 10 *μ*M) used in astrocytes whether inflexinol was treated with or without LPS (Figures 2(c) and 2(d)).

### 3.2. Effect of inflexinol on LPS-induced NO production and iNOS and COX-2 expression in RAW 264.7 cells and astrocytes

**Figure 3:**
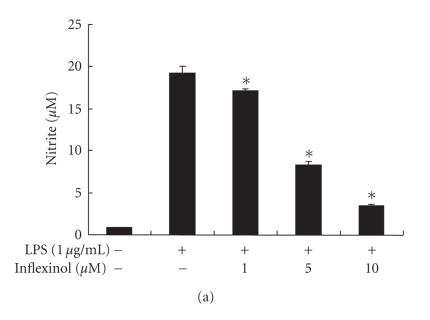

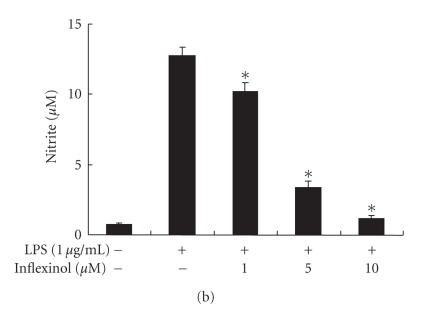

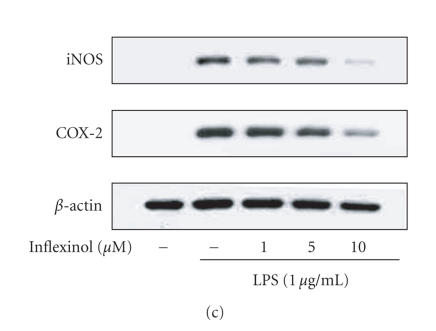

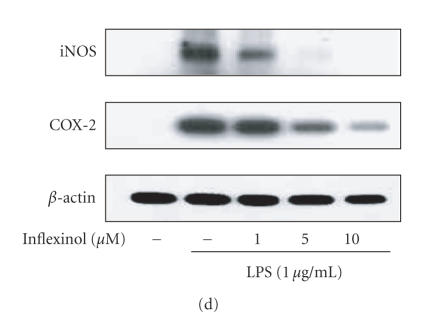
Effect of inflexinol on LPS-induced nitrite production in RAW 264.7 cells (a) and astrocytes (b). The cells were treated with 1 *μ*g/mL of LPS only or LPS plus different concentrations (1, 5, 10 *μ*M) of inflexinol at 37°C for 24 hours. NO generation was determined in culture medium as described in Section 2. The data represent the mean ± (SE) of the three independent experiments performed in triplicate. * indicates significantly different from the LPS-treated group (*P* < .05). Effect of inflexinol on the protein expression of iNOS and COX-2 in RAW 264.7 cells (c) and astrocytes (d). The cells were treated with 1 *μ*g/mL of LPS only or LPS plus different concentrations (1, 5, 10 *μ*M) of inflexinol at 37°C for 24 hours. Equal amounts of total proteins (40 *μ*g/lane) were subjected to 10% SDS-PAGE, and the expression of iNOS and COX-2 was dectected by Western blotting using specific antibodies. *β*-actin protein was used here as an internal control. Similar results were obtained from at least three different sets of experiment.

We have examined the inhibitory effect of inflexinol on NO production of RAW 264.7 cells and astrocytes induced by LPS (1 *μ*g/mL). To evaluate the effect of inflexinol on NO production in LPS-induced RAW 264.7 cells and astrocytes, nitrite accumulation was examined by the Griess assay. After co-treatment with LPS and inflexinol (1, 5, 10 *μ*M) for 24 hours, LPS-induced nitrite concentrations in the medium were decreased remarkably in a concentration-dependent manner. In RAW 264.7 cells (Figure 3(a)) and astrocytes (Figure 3(b)), the IC_50_ values of inflexinol on inhibiting LPS-induced NO production were 3.43 *μ*M and 2.66 *μ*M, respectively.

To investigate whether inflexinol inhibits the NO production via inhibition of corresponding gene expression, we determined iNOS expression by Western blot analysis. We also determined COX-2 expression since iNOS can be modulated by COX-2. As shown in Figures 3(c) and 3(d), the cells expressed extremely low levels of iNOS and COX-2 protein in an unstimulated condition. However, iNOS and COX-2 protein expression was markedly increased in response to LPS (1 *μ*g/mL) after 24 hours. Treatment with inflexinol (1, 5, 10 *μ*M) caused concentration-dependent decreases in LPS-induced iNOS expression in RAW 264.7 cells (Figure 3(c)) and astrocytes (Figure 3(d)). This result is consistent with the profile of the inhibitory effect of inflexinol on NO production. A similar inhibitory effect of inflexinol on the LPS-induced COX-2 expression was found (Figures 3(c) and 3(d)).

### 3.3. Effect of inflexinol on NF-*κ*B luciferase activity

**Figure 4:**
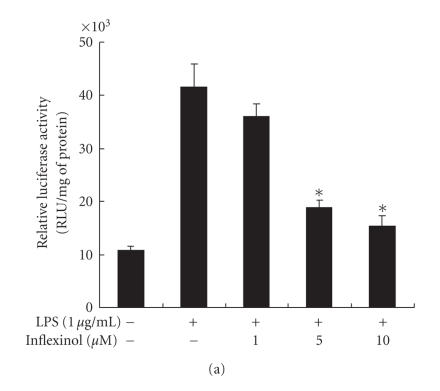

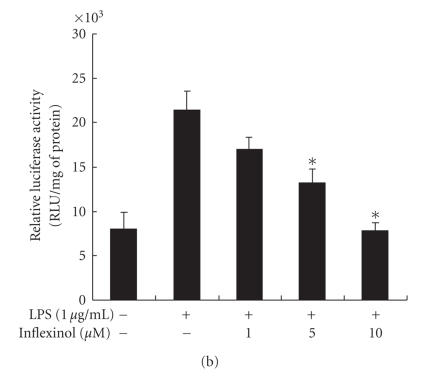

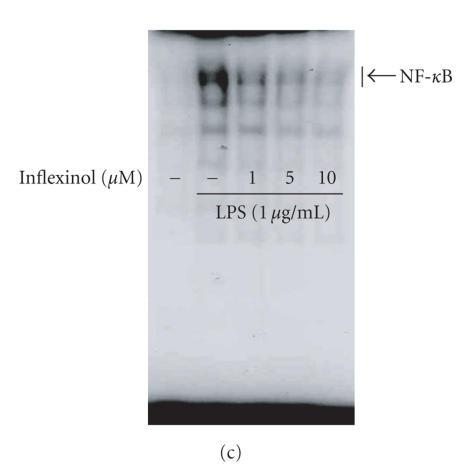

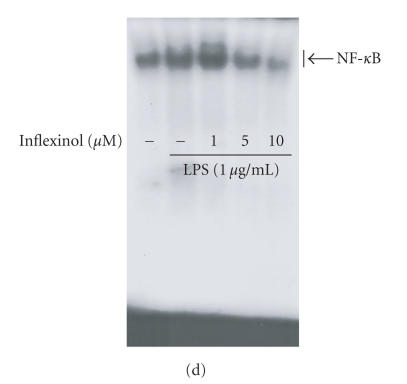
Effect of inflexinol on LPS-induced NF-*κ*B-dependent luciferase activity in RAW 264.7 cells (a) and astrocytes (b). RAW 264.7 cells and astrocytes were transfected with p-NF-*κ*B-Luc plasmid (5× NF-*κ*B), and then treated with LPS (1 *μ*g/mL) alone or with inflexinol (1, 5, 10 *μ*M) for 37°C for 8 hours. Luciferase activity was then determined as described in Section 2. Values represent the mean ± (SE) of three independent experiments with triplicate, and each luciferase activity was calibrated by amount of protein. * indicates significantly different from the LPS-treated group (*P* < .05). Effect of inflexinol on LPS-induced NF-*κ*B DNA binding activity in RAW 264.7 cells (c) and astrocytes (d). The activation of NF-*κ*B was investigated using EMSA as described in Section 2. Nuclear extracts from RAW 264.7 cells and astrocytes with LPS alone (1 *μ*g/mL) or with inflexinol (1, 5, 10 *μ*M) were subjected to DNA binding reaction with ^32^P end-labeled oligonucleotide specific to NF-*κ*B. Specific DNA binding of NF-*κ*B complex is indicated by an arrow. Similar results were obtained from at least three different sets of experiment.

NF-*κ*B controls the expression of enzymes including iNOS and COX-2 whose products contribute to the pathogenesis of the inflammatory process [20]. To investigate whether inflexinol is able to attenuate LPS-induced NF-*κ*B-mediated promoter activity, we used a luciferase reporter gene expressed under the control of five *κ*B cis-acting elements. RAW 264.7 cells and astrocytes were transiently transfected with the NF-*κ*B-dependent luciferase reporter construct according to manufacture's specification (Invitrogen), and then cell treated with LPS (1 *μ*g/mL) or cotreated with LPS and inflexinol for 8 hours. Treatment of RAW 264.7 cells (Figure 4(a)) and astrocytes (Figure 4(b)) with inflexinol resulted in a dose-dependent suppression of luciferase activity induced by LPS. In RAW 264.7 cells and astrocytes, the IC_50_ values of inflexinol on inhibiting LPS-induced luciferase activity were 2.77 *μ*M and 3.88 *μ*M, respectively. These doses inhibiting NF-*κ*B luciferase activity were similar to the doses inhibiting NO production.

### 3.4. Effect of inflexinol on NF-*κ*B DNA binding activity

Because activation of NF-*κ*B is critical for induction of both COX-2 and iNOS by LPS or other inflammatory cytokines, we determined whether inflexinol might suppress NF-*κ*B activation in LPS-activated RAW 264.7 cells and astrocytes. To investigate whether inflexinol can also inhibit NF-*κ*B activation, RAW 264.7 cells and astrocytes were cotreated with LPS and inflexinol for 60 minutes and 90 minutes, respectively, which it is the time to activate NF-*κ*B maximally of its LPS treatment (data are not shown). Nuclear extracts from cotreated cells were prepared and assayed NF-*κ*B DNA binding by EMSA. In RAW 264.7 cells (Figure 4(c)) and astrocytes (Figure 4(d)), LPS induced a strong NF-*κ*B binding activity, which was markedly inhibited by cotreatment with inflexinol in a dose-dependent manner.

### 3.5. Effect of inflexinol on LPS-induced p50/p65 translocation and degradation of I*κ*B

**Figure 5:**
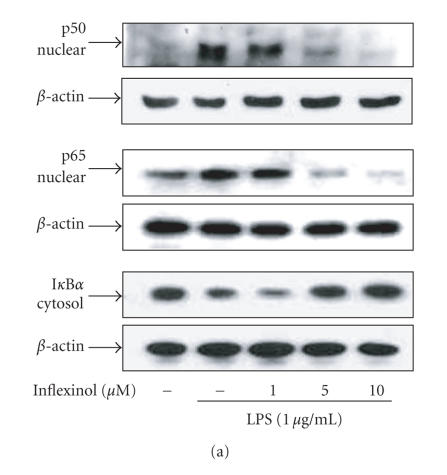

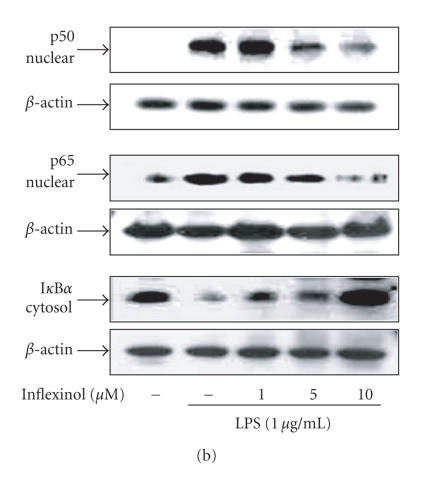
Effect of inflexinol on LPS-induced NF-*κ*B activity and degradation of I*κ*B in RAW 264.7 cells (a) and astrocytes (b). The cell treated with 1 *μ*g/mL of LPS only or LPS plus diffent concentrations (1, 5, 10 *μ*M) of inflexinol at 37°C for 1 hour. Equal amounts of total protein (40 *μ*g) were subjected to 10% SDS-PAGE. Nuclear translocation of p50 and p65, and degradation of I*κ*B were detected by Western blotting using specific antibodies. *β*-actin protein was used here as an internal control. Similar results were obtained from at least three different sets of experiment.

It has been demonstrated that LPS activates NF-*κ*B transcription factor that leads to the induction of the expression of many immediate early genes [21]. To clarify the inhibitory mechanism of action of inflexinol for LPS-induced NF-*κ*B, translocation of p50 and p65 as well as I*κ*B*α* degradation were examined. Treatment with LPS increased nuclear translocation of p50 and p65. In the presence of inflexinol, nuclear translocation of p50 and p65 was inhibited in a dose-dependent manner in RAW 264.7 cells and astrocytes. Moreover, inflexinol inhibited the LPS-induced degradation of I*κ*B*α* (Figures 5(a) and 5(b)). These results indicate that inflexinol may inhibit the LPS-induced activation of NF-*κ*B via an inhibition of I*κ*B*α* degradation as well as a translocation of p50 and p65 into the nuclear, and this effect may result in the inhibition of the LPS-induced NO production as well as iNOS and COX-2 expression.

## 4. DISCUSSION

Inflammatory processes play a critical role in the pathogenesis of many human diseases. Macrophage overproduction of inflammatory mediators such as cytokine and NO has been implicated in inflammatory diseases such as rheumatoid arthritis, septic shock, cerebral malaria, and autoimmune diabetes [22]. Astrocytes play a key role in regulating aspects of inflammation in the central nervous system. Several enzymes, such as the iNOS or COX-2, along with different inflammatory mediators such as the free radical NO or proinflammatory cytokines, have been proposed to be involved in the cell damage associated with neuroinflammation [23]. In this study, we investigated the inhibitory effects of inflexinol on LPS-induced NO production and expression of iNOS, COX-2 in RAW 264.7 cells and astrocytes. Inflexinol (1, 5, 10 *μ*M) that significantly inhibited LPS-induced NO production in a dose-dependent manner. Inflexinol strongly inhibited LPS-induced NO production in RAW 264.7 cells and astrocytes with IC_50_ values of 3.43 *μ*M and 2.66 *μ*M, respectively. These inhibitory effects may not be related with their cytotoxic effects since no effects on cell viability were observed at the concentration up to 10 *μ*M in RAW 264.7 cells and astrocytes. Comparison with IC_50_ value of indomethacin (53.8 *μ*M) and lornoxicam (65 *μ*M) being known as nonsteroidal antiinflammatory drugs in LPS-stimulated RAW 264.7 cells indicates that inflexinol has superior effects on inhibition of NO production [24, 25].

This inhibitory effect of NO production could be related with gene expression of iNOS since inflexinol inhibited iNOS protein in RAW 264.7 cells and astrocytes. Inflexinol also inhibited LPS-induced COX-2 expression. These results showed that inflexinol could interfere LPS-induced signaling involving the production of proinflammatory molecules. However, our data showed that the expression of COX-2 was found to be less sensitive than that of iNOS to the inflexinol. This could account for the higher sensitivity of iNOS gene transcription toward the inflexinol compared with that of COX-2. In fact, structurally different diterpenoids displayed differential inhibition of iNOS and COX-2 expression even though the DNA binding activity of NF-*κ*B is similar [26]. Since LPS-induced iNOS and COX-2 expression is primarily regulated by NF-*κ*B, we examined the effect of inflexinol on LPS-induced activation of NF-*κ*B using an NF-*κ*B reporter system as well as DNA binding activity using EMSA. Consistent with the inhibitory effect on iNOS and COX-2 expression, inflexinol decreased NF-*κ*B transcriptional activity in RAW 264.7 cells and astrocyte with IC_50_ values of 2.77 *μ*M and 3.88 *μ*M, respctively. Inflexinol also inhibited NF-*κ*B-specific DNA binding activity dose dependently. At the gene level, the expression of iNOS is largely regulated by transcriptional activation. The promoter of the iNOS gene contains two major discrete regions synergistically functioning for binding of transcription factors: one for NF-*κ*B, which is mainly activated by LPS [21]. There is also one NF-*κ*B consensus DNA sequence within COX-2 promoter. Therefore, inhibition of NF-*κ*B activation could contribute to the inhibitory effect of inflexinol on iNOS and COX-2 expression.

Several studies have shown that antiinflammatory agents inhibit activation of NF-*κ*B via prevention of I*κ*B degradation. I*κ*B*α* specifically binds and masks the nuclear translocation signals of p50 and p65, thereby preventing the nuclear translocation of the NF-*κ*B heterodimer. Hehner et al. have showed that sesquiterpene lactones prevented the induced degradation of I*κ*B*α* and I*κ*B*β* by diverse stimuli and therefore interfered with a common step in the signaling cascade leading to the activation of NF-*κ*B [27]. Lee et al. have also demonstrated that prevention of I*κ*B degradation by 2′-hydroxycinnamaldehyde contributed to inactivation of NF-*κ*B (p50) in antiinflammatory reaction in RAW 264.7 cells [28] as well as TNF-*α*-treated colon cancer cell death [29].

The way that inflexinol can interfere with NF-*κ*B activation is not clear. However, it is noteworthy that inflexinol contains an *α*-methylenecyclopentanone moiety as a commom functional group which is known to react with nucleophiles, especially cystein sulfhydryl groups in protein, by a Michael-type addition. A C-20-nonoxygenated-*ent*-kaurane diterpenoid (kamebakaurin, KA), isolated from *Isodon japonicus*, was suggested to interact with cysteine of DNA binding domain of the p50 subunit of NF-*κ*B [15]. Recently, KA was found to be able to interact with both p50 and p65 subunits of NF-*κ*B [26]. Hehner et al. also demonstrated that sesquiterpene lactones interfered with the activation of NF-*κ*B by preventing a degradation of I*κ*B*α* and I*κ*B*β* but lacking either the lactone or the exomethylene group in the *α*-position to the lactone function displayed no inhibitory effect on pathway leading to the activation of NF-*κ*B [27]. Similarly, the exomethylene group of inflexinol may be essential for the covalent modification via interaction with cysteine as above compounds. Moreover, data presented herein show that inflexinol significantly inhibits NF-*κ*B activation by reducing the degradation of I*κ*B. Kwok et al. have reported that exocylic methylene of sesquiterpene lactone parthenolide is required for in vivo and in vitro antiinflammatory activity and modification of cysteine 179 of IKK*β* has been proposed to mediate the pathological effects of arsenite and parthenolide [30]. Therefore, our data suggest a possibility that inflexinol inhibit the upstream proteins of I*κ*B such as IKK or 26s proteasome. This issue is being currently investigated.

On the basis of the current results and those of other reports, we propose that inflxinol inhibit the expression of iNOS, COX-2, and NO production by inhibition of NF-*κ*B DNA binding activity and transcriptional activation through prevention of I*κ*B degradation, and suggest that inflexinol may be useful as an antiinflammatory agent.
